# A general theory of consciousness I: *Consciousness and adaptation*

**DOI:** 10.1080/19420889.2020.1713967

**Published:** 2020-01-30

**Authors:** Abraham Peper

**Affiliations:** Department of Biomedical Engineering & Physics, Academic Medical Centre, University of Amsterdam, Amsterdam, The Netherlands

**Keywords:** Consciousness, cognition, adaptation, regulation, sensory images, language, autism, visual thinking

## Abstract

This paper examines how cognitive processes in living beings become conscious. Consciousness is often assumed to be a human quality only. While the basis of this paper is that consciousness is as much present in animals as it is in humans, the human form is shown to be fundamentally different. Animal consciousness expresses itself in sensory images, while human consciousness is largely verbal. Because spoken language is not an individual quality – thoughts are shared with others via communication – consciousness in humans is complex and difficult to understand. The theory proposed postulates that consciousness is an inseparable part of the body’s adaptation mechanism. In adaptation to a new environmental disturbance, the outcome of the neural cognitive process – a possible solution to the problem posed by the disturbance – is transformed into a sensory image. Sensory images are essentially conscious as they are the way living creatures experience new environmental information. Through the conversion of neural cognitive activity – thoughts – about the state of the outside world into the way that world is experienced through the senses, the thoughts gain the reality that sensory images have. The translation of thoughts into sensory images makes them real and understandable which is experienced as consciousness. The theory proposed in this paper is corroborated by functional block diagrams of the processes involved in the complex regulated mechanism of adaptation and consciousness during an environmental disturbance. All functions in this mechanism and their interrelations are discussed in detail.

## 1. Introduction

In this paper, I will examine how living beings become conscious of what they have thought, their thoughts being the outcome of neural cognitive activity. My intention is to develop an overall, general, view of the mechanisms associated with thought and consciousness, rather than a description of the exact nature of the partial processes involved. The theory developed in this paper assumes that there is no basic difference between the neural mechanism of thinking in humans and in non-human animals (hereafter ‘animals’) [1–13]. Consciousness I will place in the context of one of the main mechanisms in a living organism: adaptation. I will argue that consciousness is an inherent part of the general process of adaptation by living beings to their surroundings and understanding its function and how it is accomplished, should not be too difficult as long as the human version of consciousness is excluded. Consciousness in humans is difficult to understand as it is dependent on and interwoven with verbal communication, which complicates the matter considerably. Consciousness in animals is less complicated because it is an individual entity and achieved with easier to comprehend means like visual images. Once animal consciousness is clear, the human version can be understood more easily.

In previous work, I have addressed the process of adaptation by the body to repeatedly occurring disturbances [14–21]. These papers present a mathematical model of intermittent adaptation in biological processes. The model describes the general characteristics of adaptation as this phenomenon manifests itself in the development of tolerance to drugs. The model is a non-linear, learning feedback system. It combines a fast regulator, which reduces the immediate effect of a disturbance, with a slow regulator, which minimizes the magnitude of the error in the long run. The fast and slow mechanisms are necessary components of any adaptation process and are, for instance, also reported in models of motor skill learning [22–27]. The mathematical model is based on the assumption that when processes in living organisms are disturbed, they adapt in a way that is fundamentally the same for all processes. Knowledge about adaptation in one process, therefore, teaches us about adaptation in other processes [28–32]. This allows me to use the behavior of the mathematical model of the organism’s adaptation to drugs to gain insight into the processes involved in adaptation to other environmental disturbances.

The mathematical model comprises several mechanisms which are not found in other models of adaptation but which are fundamental parts of any adaptation process as was confirmed by simulations with the model: 1. The model demonstrates that biological adaptation is not a simple feedback mechanism such as the model of homeostasis. Adaptation is much more complex than can be described by just feedback. 2. Model simulations show the characteristic decline of the rebound (or after-effect) during adaptation – the reaction following the end of a disturbance. This phenomenon cannot be explained by feedback alone as the magnitude of the rebound in adaptation depends on the history of perturbations whereas the reaction in simple feedback depends only on the current event.^a^ 3. Adaptation to a given disturbance is not based on just the properties of that disturbance, like its magnitude, but also on the properties of previous occurrences of the same disturbance. It is anticipation which determines the reaction of the body. 4. The body separately adapts to the presence of a disturbance and to the intervals between disturbances. This is necessarily the case because after adaptation to a disturbance has taken place, the situation, and as a result the body’s functioning, has fundamentally changed [see 17]. The consequence is that there exists no fixed baseline in adaptive processes, which can have important consequences for research into adaptation.

While the development of tolerance to drugs as described in previous publications is determined predominantly by automatic processes, in the present paper I will focus on the mechanisms involved in adaptive processes which are not automatic, processes that use conscious decision-making and problem-solving. I will argue that consciousness as it manifests itself in adaptation is a quality common to both animals and humans and that, although verbal consciousness expresses itself very differently, it still fits the general phenomenon of consciousness in animals.

In this paper, I will not address subjects such as deliberation and attention, which are not essential in the basic adaptation process and its collateral mechanism of consciousness. While the mechanisms at work in drug tolerance are fairly well known and have been described in detail, the function and working of consciousness is subject to many very different views and opinions. Here, I will approach the problem of consciousness in yet another way. For the sake of clarity, the next section will define the main concepts used.

## 2. Premises

**Adaptation** is the way living organisms deal with new changes occurring in their environment. In the present context, I define adaptation as the way an organism changes its functioning in order to keep performing optimally during environmental disturbances.^b^

**Thought** or cognition I will define mainly as brain activity associated with problem-solving.^c^ I assume that the cognitive process in adaptation to an environmental disturbance does not differ from other cognitive processes. To distinguish between cognition in a physical and in a nonphysical context, I will refer to the latter as *mental thought*.

**Senses** I define as transducers able to convert environmental information into neural signals. I will not go into the way the senses function. How information from within the body is addressed I will only discuss when appropriate.

**Sensory images** are blocks of data representing aspects of objects or events in a form determined by the input – output relation of the sense in question.^d^ Although the word image is a static concept, sensory information is generally dynamic in nature (see also footnote 13).

**Language** is usually defined as a means of communication. In this paper, I will use the word language as well for the different ways animals and humans express their thoughts. If this is achieved by visual means, for instance, I will use the term visual language, in contrast to the language humans use for communication, which I will call spoken language.^e^

**Consciousness** I define primarily as the way living creatures experience new sensory information.

## 3. The conscious adaptation phase

Adaptation is the way living organisms deal with new changes occurring in their environment. As such, it is a prerequisite for life: without adaptation to a changing environment a living being cannot exist. Adaptation allows an organism to keep functioning when its situation changes, which implies that it solves the problems these changes create. The process of adaptation has two extreme states: 1. The disturbance is new and there is as yet no level of adaption. 2. The disturbance occurs regularly and there exists complete adaptation. A living organism hardly ever reaches the latter state as the environment of most of its processes is in a continual flux necessitating constant action by the organism’s adaptation mechanisms. In general, adaptation mechanisms function somewhere between the two extreme states.

The development of tolerance to a drug is a good example of how adaptation develops, although much of the adaptation process has taken place in the past. During the relatively short-lasting presence of a drug, the body learns to oppose its disturbing effects. This learning process is initiated when the drug is detected by the gustatory system or by environmental clues the organism has learned to couple to the drug presence. The adaptation process may be seen as an intermittent process of learning by the organism: during each disturbance it progressively learns how to deal with the recurrent change in its internal environment to keep functioning optimally. When full adaptation is established, the organism has learned to respond as effectively as possible in the given circumstances. In automatic processes, there are established modes or structures in place to execute solutions to particular problems. In adaptation to a disturbance which is new, the organism has to learn how to deal with the new situation. A new environmental problem has to be solved via a learning process: every time the problem occurs, the organism thinks it over and finds a solution on the basis of knowledge gathered on previous occasions. That solution then becomes conscious and is executed after which its effect is evaluated (see Section 7.1 and 7.2).

The transition from the conscious phase to an automatic process is not well defined and may be gradual [33]. Generally speaking, however, the nature of automatic processes is that they are not conscious whereas a new adaptation process is always conscious, as will be discussed^f^ [33–37]. That automatic processes are not conscious does not mean that they don’t adapt. Adaptation to changes in circumstances always has to take place. For instance, a change in drug dose requires the processes involved to adapt to a new level of functioning.

## 4. How consciousness is accomplished

As stated in Section 1, neural cognitive mechanisms in humans and animals have much in common. In this paper, I will argue that cognitive processes in humans and animals are fundamentally alike as long as verbal activity is disregarded. I will substantiate this proposition below, but I should first emphasize that consciousness as discussed at this stage of the paper differs essentially from verbal consciousness in humans, where it often dominates the innate manifestations of consciousness common to all animals. Here, it is the outcome of a cognitive process about a problem an organism is confronted with, transformed into a sensory image which makes the neural process intelligible [see e.g. 38]. I will now first try to elucidate how in the theory developed here animals manage new situations and how thought, consciousness and sensory images are part of that process.

When an animal encounters an environmental situation it is not accustomed to, it will use its cognitive capabilities to solve the problems that brings about. This requires processes like analysis, problem-solving and decision-making. The outcome of this thought process – a possible solution to the problem the animal faces – is then transformed into a sensory image which is conscious: its neural cognitive activity is translated into a form which has meaning in its outside world [see e.g. 39]. Sensory images are projections of an animal’s environment onto its neural system and they constitute what an animal knows about its outside world. When an animal encounters a situation which is new, the sensory image generated by the event is experienced consciously, or rather, the experience of that sensory activity *constitutes* consciousness. This consciousness is a fundamental part of any new event in all animal life and an inseparable part of the solutions found by its thought processes (see Section 7).

For the animal to be able to apprehend the outcome of its neural cognitive activity, it has to translate this activity into known sensations: it is expressed in a sensory image. This image is conscious as it is composed of sensory images which in the past were part of conscious new situations (see Section 7.2). When thoughts are translated into sensory images, the thoughts gain the reality sensory images have for the animal. The conversion of neural cognitive activity about the state of the outside world into the way that world is experienced through the senses is what makes a thought real and understandable.

## 5. Cognition and consciousness

### 5.1. The translation process

We have seen that cognitive activity in animals becomes conscious through its translation into aspects of the outer world. However, the cognitive process is involved in two very different modes of operation: it is part of physical actions as well as of mental thought. The cognitive process in the two situations is not fundamentally different. What is different is that the processes involved in physical actions determine the images used when cognitive activity is translated into sensory images: these images are a constituent part of the action (see Section 7). In nonphysical action, mental thought, there are no such restraints and arbitrary images are a common part of the translation process (see Section 7.5).

When the cognitive activity is part of a physical action, the conversion mechanism will select automatic processes which can execute the cognitive plan or task. It will select those processes from a fast database of relations between cognitive activities on the one hand, and automatic processes, the images which incite those processes (primary images) as well as images of these processes in action (secondary images), on the other, augmented by to the process parameters which control the functioning of those processes in the given situation (see Section 7.1). The translation provides a conscious image that is composed of the secondary images of those processes which together will execute the cognitive task.

If the cognitive action is mental only, the translation process makes use of an additional database of relations between mental activity and arbitrary images.^g^ Analogous to the expression of cognitive activity in terms of verbal images – words – I will call the expression of cognitive activity in terms of arbitrary images a language: an animal with only tactile senses will use tactile language; an animal with sight will use visual language.

It should by now be clear that the capacity to translate cognitive activity into consciousness through sensory images is a faculty universally present in human and animal life [see e.g. 40, 41]. In lower organisms – like those on cell level – the processes which establish ways to counteract the effects of environmental changes are comparable in their outcome to the cognitive process in higher organisms: they solve the problems allowing adaptation to take place. It then seems likely that the cycle described above – the expression of the solution to problems into sensory images of the way the problems are experienced – will be present there too. As all living organisms, cells then have their own, particular way of experiencing consciousness [42].

### 5.2. Consciousness in humans

Like animals, humans make their thoughts conscious by translating them into aspects of their outer world. But, for humans, that outer world consists for a large part of spoken language [10]. Whereas animals with visual senses translate their cognitive activity into visual images, which represent their outer world, humans translate their thoughts into strings of words. Spoken language, however, is not part of the innate make up of humans: visual language in animals is congenital while spoken language in humans develops after birth. When a child learns to talk, it first learns the meaning of words and their usage and only then develops consciousness in spoken language [see e.g. 43]. Verbal consciousness can only be achieved when spoken language has become the normal world for the child, when it has familiarized itself with the verbal world it is going to live in. This can be problematic for the child as this artificial, ever-changing, man-made construction [10] lacks the natural quality of the environment experienced through the senses.

Alongside their visual language, animals with sight use tactile and other languages. Likewise, humans use their senses to become conscious of their thoughts, prior or additional to verbal consciousness. There are feelings and visual images participating in the consciousness process and nearly every activity develops its own way of generating consciousness of the accompanying thoughts through sensory sensations. A tennis player thinks in ‘tennis’, a football player in ‘football’, and so on. When a tennis player hits a ball, he does not think in spoken language; he thinks in all the facets of the tennis game. His thoughts about how to hit the ball are expressed in the feeling of the racket while moving it, in the sight of the ball coming to him, in the feeling of the ball colliding with the strings on his racket, in images of the ball hitting the opponent’s side of the court, etc. His thoughts about playing tennis are translated into aspects of his game by which they become conscious because they are a known part of his outside world. These conscious thoughts are then effectuated when he hits the ball [see also 44]. His consciousness cannot reside in spoken language as the multiple tasks he is performing when preparing and then hitting the ball are too numerous and certainly too fast to be expressed in the slow and restricted means of spoken language. Every skilled tennis player knows the feeling of how he is going to hit a ball: all the forces, movements and adjustments are felt just before he actually hits the ball. They are the thoughts he has about performing a particular hit, transformed into consciousness by expressing – translating – them into aspects of his outside world, the tennis game. The same goes for professional or otherwise highly skilled players of any other fast sport [45–48] and in fact for every fast skilled activity humans engage in.

For slow sports, spoken language does play a role, but there it is restricted to general observations and considerations. When a snooker player contemplates his next shot, he thinks his possibilities over in spoken language. But when he hits the ball with his cue, the exact way of moving the cue, its speed and direction, the striking of the ball with the cue at the exact point, not a fraction of a millimeter lower or higher or to the left or right on its surface, all are determined not with spoken language but with what is generally known as ‘feel’. He feels the exact way he should hit the ball with his cue and this feeling is the conscious outcome of his thoughts about the subject [25]. His brain performs enormously complex computations on the basis of all the data about the game it has knowledge of after which the outcome becomes known to the player in feelings of the shot he is going to play: the translation of his thoughts about the matter into the way he experiences his outside world, the snooker game.

## 6. The language problem

### 6.1. Visual language in children

Mental thoughts in animals become conscious by a transformation of their neural cognitive activity into sensory images of their outside world. Animals with sight predominantly think visually; they use visual images to translate their thoughts into consciousness. Visual language is an instrument used by all animals with sight and is in all probability innately present, developing further after birth: the animal learns to associate the outcome of new cognitive efforts with visual images. There is ample evidence that the cognitive functioning of young children has much in common with that of animals [49–56]. At the same time it is clear that pre-language children think and reason [see e.g. 57]. It thus seems likely that young children, like animals with sight, use their innate visual capabilities for thinking before they learn spoken language.

This visual language a young child uses is supplemented with spoken language when the child grows older. Then an important event happens: the visual language it has been using by nature is substituted by spoken language. When spoken language takes over as the major means of making thought conscious, the visual language the child had been using largely loses that function or disappears altogether. However, this process does not take place in all children. Many keep their visual language alongside their spoken language, often making the use of spoken language problematic. Their visual thinking interferes with the spoken language and may lead to dyslexia or autism. In both these conditions, visual thinking is known to be strongly present [58–67]. In the case of dyslexia, the child has problems with utilizing spoken language; in autism spoken language is partially or sometimes completely rejected. It seems likely that both conditions are at least in part caused by the visual language competing with the new form of thinking. The process of interference causing symptoms of autism may in fact begin as soon as verbal communication develops, with the child dropping out of the learning process to hold on to its private, visual world with a consequential under-development of social skills, characteristic of autistic children [68]. A well-known example of dyslexia we find in Einstein [69–72], who thought completely visually. If he had to communicate his thoughts to others, he had to translate the visual images of his thought into spoken language, something he found hard to do.^h^ Many people are in a state somewhere between harmless forms of dyslexia and a more or less complete rejection of spoken language while many continue to use some form of visual language without much effect upon their verbal functioning (see Roger Penrose [73]: *The Emperor’s New Mind*).

Despite the fact that visual thinking is found regularly, notably, but not solely, in those with autistic traits, for completely verbal thinkers visual thinking generally appears incomprehensible. Conversely, in spite of using language for communication themselves, visual thinkers find it difficult to imagine that somebody can become conscious of his thoughts by translating them into words.

### 6.2. Spoken language versus visual language

Compared with spoken language, thinking through visual images is a very refined and accurate method of translating thought into consciousness while it can be extremely fast. Spoken language is one-dimensional and very time-consuming: words are placed in a time sequence to form a composition with a certain meaning. Visual thinking provides an incomparably more detailed representation of a thought as the images are two- and often three-dimensional and can contain countless detail and meaning while the spatial composition in itself provides meaning and nuance. Spoken language is very much restricted because it has to be intelligible to others on account of its function in communication. Visual language can be constructed in any way its user wants as it is an individual, private quality. Due to its nearly unlimited complexity, visual language lacks the vagueness of spoken language and is therefore completely superior, but for the missing component: communication with others.^i^

## 7. Elaborations on the theory

### 7.1. Adaptation to environmental disturbances

#### 7.1.1. The adaptation process

**Figure 1.**
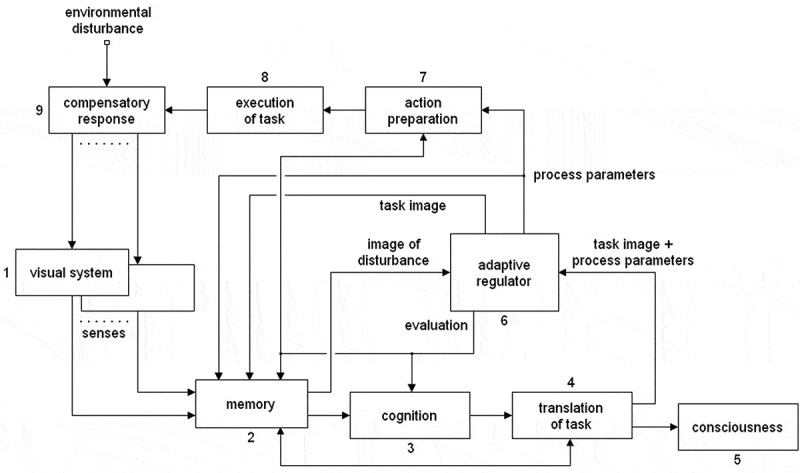
Functional block diagram of the processes involved in adaptation and consciousness during an environmental disturbance. The arrows indicate the interactions between the functions

While mental thought is confined to cognitive activity, the cognitive action in an adaptation process during an environmental disturbance is part of a complex response to the situation. The main object of adaptation is to actuate and organize processes which can oppose the disturbance.^j^ A diagram of the functioning and interactions of the processes involved when an organism adapts to a new environmental situation is shown in Figure 1, Section 7.2. In the diagram, the blocks show the role of the processes participating in adaptation as autonomous functions and I will refer to them as such. However, they should not be thought of as modules.

In Section 7.2, the block diagram will be discussed in detail. In the present section, I will try to elucidate the general features of the adaptation process. The adaptation mechanism is a regulated system, which means that it is a feedback loop. As all activity in a closed feedback loop is interrelated, it is not really possible to predict the effect of an action somewhere in the loop on the loop’s total functioning, other than with a mathematical model [17,18]. In the usual qualitative approach, not more than a general impression of a regulated system can be given and the conclusions are often not accurate and regularly completely wrong. In the block diagram of Figure 1, the regulation of the adaptive system is determined by the adaptive regulator. This adaptive regulator is basically the mathematical model developed in previous work [see e.g. 17, 18] and all conclusions derived there are applicable to the present situation.

Adaptation is an additive mechanism: it solves environmental problems by counteracting them [see 15]. This implies that its opposing action is of the same nature and develops in the same way as the disturbance. This can be observed clearly in motor adaptation, a much investigated subject of research in this field. A well known and applicable example is given by Shadmehr & Mussa-Ivaldi [74],who used a force field to disturb the reaching movements of a subject. After adaptation, the subject had learned to oppose the force field by exerting the same force in the opposite direction in order to restore normal operation. The block diagram of Figure 1 is especially applicable to adaptation processes such as those caused by disturbances of movement as discussed in the study by Shadmehr & Mussa-Ivaldi.

#### 7.1.2. The general structure of the adaptive system

Obviously, the functioning of the senses is pivotal in the adaptation process. The senses provide information in the form of images.^k^ These sensory images are stored in special memory units. A memory unit contains all information about the process a sensory image activates.^l^ The incoming (primary) images are analyzed to discriminate between new images and images which are already known to the organism and which are the result of earlier experiences or passed on innately. Known sensory images activate automatic, non-conscious, processes while new sensory images activate conscious adaptation processes.

The process of adaptation to environmental disturbances comprises two distinct mechanisms: a cognitive system, which seeks optimal solutions to problems that are new to the organism, and a regulatory system. This adaptive regulator is a mechanism universally used by living organisms to suppress the effects of disturbances they are confronted with. It is summarized in Section 1 and extensively discussed in previous publications, where it is part of a mathematical model of drug tolerance [see e.g. 17, 18]. The adaptive regulator discussed in the model of adaptation to drugs regulates one parameter only: the magnitude of the compensating response to the drug action. The adaptive regulator in the present situation is much more complex. The subject of the regulation are all the parameters determining the behavior of the processes involved. The parameter values are regulated to obtain overall adaptation and are then stored in the memory units referred to above.

When a change in environment occurs, the cognitive mechanism will contrive a plan to counteract its effect on the organism. The realization of this plan, the task the system will try to accomplish, is organized by the translation mechanism described in Section 5. It will collect from memory the automatic processes and their parameter values which together can execute this task. The task image – an image composed of the secondary images of the processes involved – shows what their effect will be. If the plan is sound and the translation accurate, the task image will match the image of the disturbance. The information about the task will subsequently be passed on to the adaptive regulator and will be implemented and executed (see Sections 7.1.5 and 7.2). The task image shows the task as it is supposed to be executed in order to oppose the disturbance. The relationship between the image of an action and its execution is well known from the literature [44,74–83].

#### 7.1.3. The conscious image

The image of the action the organism has planned – the task image or secondary image – is experienced as conscious, as discussed above. Such an image is conscious as it is composed of conscious images of previous adaptation processes. The representation of the task by secondary images is what makes the cognitive process conscious: I experience how I will hit a ball in tennis before I actually execute hitting the ball. These sensations are secondary images stored during previous experiences I had when hitting a ball. Secondary images are central in the functioning of the adaptive regulator, as will be discussed in Section 7.2. They show the functioning of automatic processes after their last adaptation cycle.^m^ Secondary images are stored in memory units which define the activation and functioning of automatic processes, as indicated above. A secondary image is coupled to the primary image – the sensory image which activates the adaptation process – and to the parameter values determining the functioning of the process.

The conscious task image shows the plan the organism has developed for the execution of the outcome of its thought process, while at the same time this image shows how the organism will perceive the execution of that plan. Apparently, the outcome of the neural cognitive activity, the task which is to be executed, is translated into an image the senses would provide if they were to observe the outcome of this task (see also 84,85). This close relationship between sensory information and the executed task is a consequence of adaptation being a feedback mechanism. In such a closed regulation loop, all activity is interrelated (see Section 7.1.1).

#### 7.1.4. Inaccuracies of the adaptation process

The general process of adaptation has to cope with several problems. First there is the noise, present in any sensory system, which adds uncertainty about the nature, the moment of occurrence and the time course of the information received. This may cause the adaptation process to produce inaccuracies or even an erroneous outcome. But there exist more fundamental problems. One important problem is that adaptation to a certain disturbance does not merely mean that the organism knows how to cope with that particular disturbance, but that it knows how to cope with that disturbance having a certain *magnitude* [see 17]. However, the magnitude of a disturbance can only be known to the organism after the disturbance has ended. Whenever a disturbance occurs, the organism will therefore have to estimate its magnitude, which it must then base on the information available: the magnitude of previous occurrences of that particular disturbance. But this means that a change in magnitude of the disturbance may result in a large change in its effect, followed by a period of incomplete adaptation [see 17]. An immediate and accurate reaction to a disturbance is apparently not possible. If an organism could react to a stimulus in a rapid and appropriate manner, adaptation would be instantaneous. As it is, an organism only slowly learns the true nature and magnitude of a disturbance and its consequences [see e.g. 17,86]. That living organisms base their behavior to a large extent on experience and anticipation can clearly be seen in the way they deal with drugs: their reaction to a drug does not depend on the dose of an administered drug only, but as much on what the organism expects the dose to be [see 21].

A comparable problem is that a reaction to a disturbance may change the relation between the organism and the source of the disturbance, which in effect changes the disturbance to the organism itself [87–90]. The consequence is that, as in the problem discussed above, the adaptation process at any moment cannot be based only on the perceived environmental situation of that moment, but has to rely to a large extent on information from the past: knowledge about previous disturbances of the same nature or about the way the body’s behavior might influence the sensory information.

An additional problem for an adaptation process is that a disturbance perceived through the senses is generally part of a larger sensory image. Extracting the relevant information from these often complex and unrelated image components, composed of independently changing contents, requires a difficult cognitive process which constitutes an essential part of the adaptive operation.

Furthermore, there is the problem that the outcome of an adaptation process is a compromise. While the aim is to find an optimum solution for the organism as a whole, the outcome may be far from optimal. Adaptation processes often affect other processes in the organism not directly related to the task and it may be difficult or even impossible to find a solution which fits all (see Section 2).

Due to these difficulties, finding an acceptable solution to an environmental problem is often a laborious operation and necessitates a process of trial and error. As a result, adaptation is a slow process of working step by step toward a solution to the problem. Every step is evaluated and provides information for the next try, until the outcome is considered to be satisfactory and the process becomes automatic.

#### 7.1.5. Considerations about the execution of the task

For the implementation of the task, the individual processes which will execute the task are prepared and activated on the basis of the information from the adaptive regulator. The process parameters are set after which the task is executed. The result constitutes the counteraction to the environmental disturbance by the adaptation process.

As noted above, the primary image might show a complex scene of which the disturbance may be only a small part while the secondary image – the image of the task – shows the isolated disturbance as estimated by the cognitive process. The adaptive regulator compares the task image to the disturbance component in the primary image and then adjusts the parameter values of the processes which execute the task on the basis of this information. Although adaptation only develops to the disturbance component in the primary image, the complete image of the environmental situation determines the triggering of the specific adaptation process: adaptation to the disturbing force field in Shadmehr’s experiment does not provide adaptation to, for instance, a comparable disturbance during a game of snooker.

If the solution devised by the cognitive process is effective, the result will be a decrease in the disturbance experienced by the organism, which then shows in the primary image. If the solution is not adequate, the effect of the operation will be small or absent and a better answer to the problem has to be found. This evaluation of the quality of the cognitive outcome is a vital element in the adaptation process. Adaptation implies finding a solution to a problem and a test of the adequacy of this solution determines the efficacy of the adaptation process (see Section 7.2).

When after several regulatory cycles the organism considers the suppression of the disturbance by the compensatory response to be optimal in the given circumstances – when it is satisfactory and does not improve anymore – the adaptation phase is over and the cognitive action ends. Without cognitive action, the process loses consciousness and becomes automatic after which the task is executed on the basis of the knowledge gathered during adaptation. But even when a process has become fully automatic, the *adaptive regulator* still remains active, adapting the process parameters to changes in circumstances (see Section 7.3).

Not every cycle in the adaptation process is necessarily followed by an execution of the task [see e.g. 44, 75, 91; Jeannerod, 1995, 2001]. Whether a solution found by the cognitive process to a new environmental disturbance is executed depends on the adaptive regulator’s estimation of the quality of the outcome as described above. When a task is not considered adequate, it will not be executed. In this case, the output image of the cognitive process still becomes conscious as the course of the cognitive adaptation procedure is not different from normal. What then is absent is information about the actual effect of the task. But when, after modifications, the solution is accepted by the adaptive regulator as satisfactory, it will be executed in the usual way. This procedure allows an organism to test alternative solutions to environmental problems without consequences and energetic costs.

### 7.2. Block diagram of the processes involved in adaptation and consciousness

Figure 1 shows a functional block diagram of the processes involved in adaptation and consciousness during an new environmental disturbance. The blocks do not show neural processes but indicate the functions involved in the process of adaptation and their effects. The arrows indicate the interactions between the functions. A block diagram of automatic processes is discussed in Section 7.3.

When an environmental disturbance occurs, it induces sensory images in the *sensory systems* (1) block. For the sake of simplicity, only the visual system is mentioned explicitly with the other senses implicit, but all senses participate equally in the mechanisms discussed. In case of a new disturbance, a conglomerate sensory image – an image comprising information about the disturbance from all senses involved – is stored in a memory unit in the *memory* (2) block, the archive for data about the bodily processes. Block 2 analyzes incoming images to differentiate between new images and images related to automatic processes already in memory.

A sensory image resulting from a new environmental disturbance – the primary image – is transferred from Block 2 via its respective outputs to the *cognition* (3) block and the *adaptive regulator* (6) block, the two major functions in the adaptation process. In reaction to the new sensory stimulus, *cognition* develops a plan of how to react to the change in environment. This plan – the task the organism will try to implement – is translated by the *translation of task* (4) block into information which ultimately results in the execution of the plan.

The process of translating the cognitive outcome establishes the automatic processes which together can execute the task and it collects from memory the secondary images which represent those processes. These images are the outcome of previous adaptation processes. They have been coupled in memory to the sensory images which incited these adaptation processes and to the parameter values of the processes involved after adaptation was completed. Together, they form memory units which define the activation and functioning of automatic processes. The connection between Block 2 and Block 4 indicates the interaction between the translation process and memory. The representation of the cognitive output by secondary images is what makes the cognitive process conscious, a function represented by the *consciousness* (5) block.

The output image of Block 4 is an approximation of the solution *cognition* has found to oppose the problem imposed by a new environmental disturbance. The applicability of this solution depends on its capacity to counteract the disturbance, which in turn depends on its resemblance to the disturbance. It is determined by factors such as the availability of appropriate processes to execute the task, the quality of the neural cognitive process and the accuracy of the translation.

Central in the mechanism of adaptation to new disturbances is the adaptive regulator. Its general function is comprehensively described in previous publications [see e.g. 17, 18]. Its operation is twofold: it evaluates whether the solution *cognition* has come up with to oppose the disturbance is effective, a vital function in adaptation (see section 7.1.5),^n^ and – on the basis of the outcome of this evaluation – it adjusts the parameter values of the processes which will execute this task. During the adaptation cycles, the adaptive regulator compares the task image to the disturbance component in the primary image. Adaptation is completed when their resemblance is optimal. This procedure is an essential component of the adaptation process in its effort to obtain optimal suppression of the disturbance. As the task image is a composite of conscious secondary images, it is consciously experienced. This implies that the counteraction of a new environmental disturbance by the adaptation mechanism is fundamentally a conscious event.

The information the adaptive regulator provides about the accuracy of the solution *cognition* has found for the problem – referred to as *evaluation* in the diagram – determines the course of the adaptation process: If accuracy is low, *cognition* will try to find an alternative answer to the problem. If it is high, the adaptation process is set as finished and the output image of the adaptive regulator is stored in memory with the sensory image which initiated the adaptation process and the parameter values of the executive process as established by the adaptive regulator. The process will then be defined by the initial sensory image and the task image complemented with its parameter values. Together, they form a memory unit containing all information relevant to the process, which then loses consciousness as cognition will not be involved anymore. These memory units describe disturbances as they are experienced by the organism and the functioning of the automatic processes initiated by the disturbances.

For the implementation of the task, the individual processes which will execute the task are prepared and activated. This procedure takes place in the *action preparation* (7) block. On the bases of the information the block receives from the adaptive regulator, the parameters of the processes are set after which the task is executed in the *execution of task* (8) block. The output of Block 8 is represented by the *compensatory response* (9) block and constitutes the counteraction to the environmental disturbance by the adaptation process.

There are basically two possible configurations for the adaptive process to a disturbance and consequently two possible configurations for the block diagram: an adaptation process is caused either by a change in the environmental situation or by a change in the way the environment is conceived by the organism. Figure 1 shows the first possibility: adaptation expresses itself as a change in the effect of the disturbance. In the case of a change in the way the environment is conceived, the adaptive action should affect the transfer function of Block 1, *sensory systems*. This subject is extensively treated in previous papers on adaptation [see e.g. 17, 18].

### 7.3. Automatic processes

**Figure 2.**
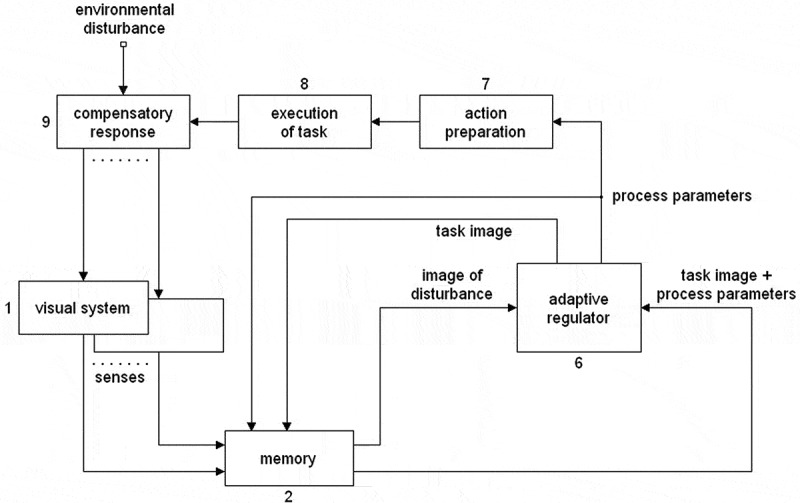
Functional block diagram of the processes involved in the initiation, and execution of automatic processes following a known disturbance. This configuration corresponds to the mathematical model of adaptation discussed in previous publications [see e.g. 17, 18]

Figure 2 shows a functional block diagram of the processes involved in the initiation and execution of automatic processes following a known disturbance. The dynamics of this configuration is addressed in detail in previous publications on a mathematical model of adaptation in drug tolerance [see e.g. 17, 18].

An automatic process is a process whose functioning is determined previously by adaptation, as pointed out above. The final outcome of the adaptation phase consists of an image of the task in its definitive form and the parameter values which describe the functioning of the physical processes involved in executing the task. This information is coupled in memory to the sensory image originating from the disturbance which initiated the adaptation process, together forming a memory unit defining that particular process.

In the configuration of Figure 2, there is a direct connection between the *stored images* (2) block and the *adaptive regulator* (6) block. This connection bypasses the cognitive and conscious functions shown in Figure 1, which are not active in automatic processes. When a sensory stimulus known to the body occurs, the image of the task and its parameter values, stored in memory, are transferred to the adaptive regulator. During the adaptation process that follows, the process parameter values are adjusted by the adaptive regulator for optimal functioning in the given circumstances. This happens in the same way as during adaptation to a new disturbance: the adaptive regulator estimates its ability to oppose the disturbance as indicated by the evaluation function. What is absent here is the cognitive part, the outcome of which was set previously by the adaptive process. The rest of the process is not different from the one discussed in Section 7.2: The latest parameter values update those in the memory unit and are then transferred to the *action preparation* (7) block. The output of Block 7, the task, is then executed by the *execution of task* (8) block. The output of Block 8 constitutes the counteraction of the adaptation process to the environmental disturbance, represented by the compensatory response (9) block.

### 7.4. Inner feelings and emotions

Just as the visual expression capability of the body induces consciousness by creating visual representations of thoughts; the interoceptive system elicits consciousness by transforming cognitive activity into interoceptive images [see e.g. 92]. Emotions are an expression of bodily states [39,93–102], but these bodily states are themselves an expression of thoughts preceding them [see e.g. 103]. How this form of consciousness manifests itself is not restricted to simple feelings like pain, hunger, warm and cold or to the place where those sensations are experienced. The interoceptive consciousness mechanism uses these and all other stored emotions to create its own image of a certain thought. They are the instruments the body uses to transform a thought into consciousness. It may, for instance, create a mixed feeling of cold and cramping pain situated in the stomach to make a thought of anxiety conscious. However, most thoughts which are expressed as inner feelings and emotions manifest themselves in a much more complex way, using a mix, a composition, of many different interoceptive sensations, all in different shades [93, 94, 100, 104,105]. In humans, where verbal consciousness strongly dominates, emotions are a clear example of how in essence arbitrary sensory functions can make cognitive activity conscious.

### 7.5. Additional considerations

Adaptation may reveal itself in two fundamentally different ways: 1. As a reaction to an environmental disturbance. 2. As part of an action taken by the organism itself. Figures 1 and 2 mainly pertain to the behavior of the body when confronted with an environmental disturbance. In this situation, adaptation is a complex event. When an action is initiated by the body itself, regulation by the adaptive regulator does not take place, which simplifies the situation. If the body initiates an action, the outcome of the cognitive activity is executed as described in Figure 1 but with the regulatory faculty of the adaptive regulator switched off. The parameter values of the processes which determine the execution of the task, determined by the translation process, are passed on unchanged. The adaptive process then takes place during a series of learning cycles. These learning cycles are conscious and when executed will use the evaluation function of the adaptive regulator for analysis of the action’s effect. The adaptive regulator then provides a measure of how well the sensory image of the executed task resembles the desired action.

If an action is evoked by an environmental disturbance, as is for instance the case in motor adaptation, the image used to represent the cognitive output is necessarily a functional part of the adaptation process; e.g. in motor action the images are kinesthetic. This is not different in actions initiated by the organism when there is a motor task to be executed – to hit the ball in a tennis service, for example. However, if the cognitive action is solely mental – for instance finding the solution to a theoretical problem – there is no physical task to be executed and the organism may use any image in a symbolic, metaphorical way to represent the outcome of the cognitive activity. Just as arbitrary sounds in human languages represent specific objects, thoughts, feelings, etc. [106, 107, 54], arbitrary images can be used in other forms of thinking. This is illustrated by visual thinking, where thoughts may be expressed in arbitrary visual images, images which in the past were associated with particular cognitive activities. In the case of thought as a purely mental operation, the adaptive regulator is not involved in any way and the evaluation of the outcome will be cognitive only. But, as stated previously, the cognitive process in mental thought does itself not differ from that in adaptation to an environmental disturbance.

An issue related to the use of arbitrary images in mental thought is the phenomenon of associative stimuli. An associative stimulus is an arbitrary sensory image or event which was coupled to a certain process in the past in a Pavlovian way by a learning process and may incite or block or in other ways influence the execution of that activity [108, 109]. Information about an associative stimulus is stored in the memory unit together with the other components of the process, as described above. Any process may be activated by an associative stimulus. The salivation of Pavlov’s dog is the best known example, but a more compelling illustration of this mechanism, discussed in previous publications, is association as it manifests itself in drug tolerance. If a drug is administered intravenously, the natural – oral – route of exogenous substances entering the body is bypassed. The normal gustatory triggering of the compensatory response then does not take place and the body has to rely on associative cues to trigger the compensating response to the drug. This cue is often the drug scene and if the drug is taken in a different setting, the usual stimulus is not present and the compensating response is not initiated. This then results in a strong increase in the effective dose and can lead to death by overdose [17,110,111].

I have not tried to elaborate on the nature of cognition, or gone into aspects of thought which are not essential in adaptation as it is discussed here, like attention or deliberation. But it might be argued that, in attention and deliberation, the object of the thought is always perceived as a more or less new experience by which the process obviously becomes conscious. This is certainly the case in verbal communication, a reason why this is always conscious, as will be discussed in part II of the paper.

## 8. Final comments

Spoken language largely shapes the world humans live in and thus largely determines their consciousness. It follows that the way spoken language articulates the thoughts of humans will have a strong effect on their consciousness. As observed above, besides spoken language, we have other means of becoming conscious of what we have thought. Thinking in feelings, for instance, is at least as conscious as is consciousness via spoken language. The feelings of happiness, anger or fear can be extremely conscious, in all probability more so than is possible in verbal consciousness. But those feelings are not communicable nor can they be transformed into spoken language. We have no way of translating a feeling of fear into spoken language and when we try, it becomes something very remote from the feeling we actually have. Our senses have equipped us with a range of different ways of becoming conscious about ourselves and the world around us, but the dominating force in our life, communication with others through spoken language, cannot express natural manifestations of consciousness in an adequate way. In part II of this paper, I will discuss in greater detail the problematic nature of verbal communication and the consequences of the use of spoken language on human consciousness. Part II will also analyze the notion of the Self in the context of spoken language and the moral aspects it entails.

## Notes

[a] In the literature, the occurrence of a reaction or after-effect after a disturbance has ended is often taken as proof that the process under scrutiny is adaptive. However, any more or less differentiating process, feedback or otherwise, shows a reaction after a disturbance has ended and only a series of after-effects with declining magnitude indicates adaptation [see e.g. 17].

[b] With regard to ‘optimal’, we do not know what criteria define optimal for an adapting organism. They are determined by deliberations too complex to understand and depend on the species, the individual, the situation, the history of the individual, etc. However, this does not mean that optimal is not a real parameter for an individual in a certain situation. A problem faced by any adapting system is that it often has to satisfy multiple, perhaps conflicting, goals [17,20,21,112,113].

[c] The words thought an cognition are here often used interchangeably as they both refer to the same neural process. The medium in which the outcome of a thought process is expressed to become conscious, however, can differ widely as will be discussed.

[d] It should be realized that an ‘image’ of the environment seen or experienced by a living organism through its senses is neural information representing that environment. This information depends on how its senses operate, i.e. on their transfer function. The relation between sensory images and environmental situations is innately acquired or develops through experience and is essentially arbitrary [13,39, 89, 90, 114]. At the same time, it is dynamically dependent on the relation of the organism with its environmental situation [see e.g. 115].

[e] The term visual language is often used to indicate a translation of verbal language into visual symbols but should then be denominated as visual-verbal language.

[f] That new adaptation processes are conscious is generally accepted, see e.g. Baars [35]: ‘The loss of consciousness of a predictable event is the signal that the event has been learned completely. Habituation of awareness is not just an accidental byproduct of learning. It is something essential, connected at the very core to the acquisition of new information. And since learning and adaptation are perhaps the most basic functions of the nervous system, the connection between consciousness, habituation, and learning is fundamental indeed.’

[g] The word database and the division into two data bases is an arbitrary description of the real, neurological process, used to elucidate the translation mechanism.

[h] Roger Penrose [73], The Emperor’s New Mind: *Letter from Einstein: ‘The words or the language, as they are written or spoken, do not seem to play any role in my mechanism of thought. The psychical entities which seem to serve as elements of thought are certain signs and more or less clear images which can be “voluntarily” reproduced and combined. ...The above mentioned elements are, in my case, of visual and some muscular type. Conventional words or other signs have to be sought for laboriously only in a second stage, when the mentioned associative play is sufficiently established and can be reproduced at will*.’ Temple Grandin [60], Thinking in Pictures: *‘Einstein told his psychologist friend Max Wertheimer, “Thoughts do not come in any verbal formulation. I rarely think in words at all. A thought comes, and I try to express it in words afterwards**’*.*”*

[i] With the possibilities digital technologies offer at present, it might be possible to develop visually based communication methods which might be faster and more accurate than verbal communication.

[j] The word *process* is used here in a very broad sense. It may mean a procedure, a function, an action, an operation or an activity of any kind such as motor functions, electrical activity, chemical reactions etc. In addition, processes are composed of sub-processes, the functioning of which often depends on parameter values set in the main process.

[k] The word ‘image’ is a visual metaphor widely used for the way information is presented by sensory systems (see Section 2). This is a confusing use of language, however: the sensory information is generally dynamic in nature, resembling more a film than an image [116, Land et al., 2013]. This is especially the case in kinesthetic data and when data from different senses is presented. It might be more accurate to regard images in this respect as maps of data, either static or dynamic [Singer, 2015].

[l] All applications of automatic processes have their own data storage. That this is the case should be clear: the automatic use of a certain muscle in tennis is different from the use of the same muscle in, say, snooker.

[m] A secondary image shows an approximation by *cognition* of the disturbance in the initial primary image. After a successful adaptation procedure, the disturbance in the primary image is suppressed but is then accurately shown in the secondary image.

[n] The adaptive regulator published previously [17,18] makes use of a cross-correlation function to determine the magnitude of the component in the sensory signal which matches the solution *cognition* has developed to counteract the disturbance. This approach works very well in the mathematical model described there. It is a matter of speculation whether this method is also utilized in actual biological adaptation processes; there may be other or better methods.
